# The Effects of an Educational Program on the Professional Quality of Life and Health of Nurses: A Cluster Experimental Design

**DOI:** 10.1097/JNR.0000000000000426

**Published:** 2021-03-19

**Authors:** Chia-Yun FU, Chia-Chan KAO, Ruey-Hsia WANG

**Affiliations:** 1 PhD, RN, Assistant Professor, Department of Nursing, Fooyin University, Kaohsiung, Taiwan, ROC; 2 PhD, RN, Associate Professor, Department of Nursing, I-Shou University, Kaohsiung, Taiwan, ROC; 3 PhD, RN, Professor, College of Nursing, Kaohsiung Medical University, Kaohsiung, Taiwan, ROC, and Adjunct Researcher, Department of Medical Research, Kaohsiung Medical University Hospital, Kaohsiung, Taiwan, ROC.

**Keywords:** nurses, professional quality of life, physical health, mental health, compassion fatigue

## Abstract

**Background:**

The complexity of the healthcare environment and intense workloads may negatively impact the health and professional quality of life (ProQOL) of nurses. Prior research has identified a significant association in nurses between ProQOL and health. Developing an intervention to improve the ProQOL and health of nurses may benefit the quality of nursing care.

**Purpose:**

The aim of this study was to explore the effects of a compassion fatigue Resiliency, mindfulness Respiration, and Relatives and friends’ support (i.e., 3Rs) educational program on ProQOL, physical health, and mental health in nurses.

**Methods:**

A cluster experimental design was used in this study to recruit registered nurses at two regional teaching hospitals in southern Taiwan as participants. The experimental group (*n* = 67) attended the 4-week (2-hours-per-week) 3R educational program. The control group (*n* = 57) received no intervention. The outcome variables, including compassion satisfaction, burnout, secondary traumatic stress, physical health, and mental health, were measured at baseline, at the end of the intervention (immediate effect), at 4 weeks postintervention (short-term effect), and at 12 weeks postintervention (medium-term effect). The study was conducted from May 2017 to December 2017.

**Results:**

Increases in compassion satisfaction and mental health and decreases in secondary traumatic stress were significantly greater in the experimental group than in the control group between baseline and all three posttest time points. Moreover, burnout decreased and physical health improved more significantly in the experimental group than in the control group between the baseline and end of intervention time points (*p* < .001).

**Conclusions/Implications for Practice:**

The 3R educational program intervention, integrating compassion fatigue resiliency, mindfulness respiration, and support from relatives and friends, had immediate and positive effects on ProQOL as well as physical and mental health. Moreover, the intervention was shown to have short-term and medium-term positive effects on compassion satisfaction, secondary traumatic stress, and mental health. Nursing managers may apply programs that integrate compassion fatigue resiliency, mindfulness respiration, and relatives and friends’ support to improve ProQOL and health in nurses.

## Introduction

The complexity of the healthcare environment and the intensity of workloads may impact negatively on the physical and mental health of nurses, causing skeletal and muscle pain, poor sleep quality, depression, and other effects (Freimann et al., 2016; Li et al., 2019; Weigl et al., 2016). Poor physical and mental health in nurses may lead to increases in sick leave requests and patient safety-related problems (Carlesi et al., 2017; Van Gerven et al., 2016). Improving the physical and mental health of nurses is a crucial issue for nursing managers. Professional quality of life (ProQOL), defined as the quality perceived by professional providers when performing work (Stamm, 2010), includes the two aspects of compassion satisfaction and compassion fatigue. Compassion satisfaction, the positive aspect of ProQOL, is the satisfaction derived from helping others (Jang et al., 2016). Compassion fatigue, the negative aspect of ProQOL, consists of burnout and secondary traumatic stress and fosters negative feelings toward clinical work and contributes to physical and mental exhaustion (Nolte et al., 2017; Ruiz-Fernández et al., 2020). Burnout is a negative emotional reaction to stressful work environments (Rayan et al., 2019), whereas secondary traumatic stress is caused by deep, vicarious traumatization associated with caring for patients (Peters, 2018). Levels of burnout and secondary traumatic stress have been found to be higher in nurses than other health practitioners, such as physicians, social workers, and palliative care professionals (Cavanagh et al., 2020). Intensive care unit nurses have been found to experience higher levels of burnout and secondary traumatic stress than either ward nurses (Kawar et al., 2019) or oncology nurses (Mooney et al., 2017). Higher compassion satisfaction has been significantly associated with better physical and mental health (Fu et al., 2018), whereas higher levels of burnout and secondary traumatic stress have been significantly associated with poor physical and mental health (Fu et al., 2018; Ruiz-Fernández et al., 2020). Improving ProQOL may benefit the physical and mental health of nurses.

### Background

Strategies for improving ProQOL have been previously developed. Compassion fatigue resiliency refers to helping professionals enhance their resilience against compassion fatigue and, ultimately, enhance their empathy toward patients, themselves, and their peers (Figley & Figley, 2017). Gentry et al. (2002) proposed a compassion fatigue resiliency program that includes five components, as described in the following: (a) self-regulation: use muscle relaxation to activate the parasympathetic nervous system and learn to change negative perceptions into positive ones, thereby strengthening recovery ability, resiliency, and adaptability of stress; (b) intentionality: modify impulsive thinking and negative stress-related coping behaviors and cultivate the habit of thinking thoroughly before reorganizing oneself for work; (c) perceptual maturation/self-validation: change one’s mood, relax when encountering stress, identify with one’s own response to stressful events through self-talk, and format work-related negative feelings into normal requirements; (d) connection and support: cope with stress through interactions with an organization or support network; and (e) self-care and revitalization: balance physical and mental health by doing aerobic exercise, following appropriate dietary and sleep regimens, and strengthening professional abilities.

A compassion fatigue resiliency intervention has been shown to significantly increase compassion satisfaction, with burnout and secondary traumatic stress significantly decreased at the end of intervention (Flarity et al., 2013). Furthermore, two longer follow-up studies found significantly decreased secondary traumatic stress at 2 months (Flarity, Nash, et al., 2016) and 6 months after this intervention (Flarity et al., 2018). These findings support that compassion fatigue resiliency interventions improve compassion satisfaction, burnout, and secondary traumatic stress. Nevertheless, the effect of this compassion fatigue intervention on physical and mental health in nurses has not been investigated adequately.

“Mindfulness” refers to paying attention purposefully, being in the present moment, and nonjudgmentally unfolding experiences from moment to moment (Kabat-Zinn, 1982). Mindfulness may be achieved in a variety of scenarios, including eating meditation, breathing exercises, and meditation exercises, to activate the parasympathetic nervous system and reduce negative thinking to achieve physical and mental balance (Killingsworth & Gilbert, 2010). Mindfulness may also reduce the production of stress hormones, such as α-amylase (Duchemin et al., 2015). In addition, studies have reported significantly decreased levels of burnout and secondary traumatic stress at the conclusion of mindfulness interventions (Duarte & Pinto-Gouveia, 2016). Although meditation and breathing exercises have been shown to significantly increase levels of compassion satisfaction and significantly decrease levels of burnout and secondary traumatic stress at the conclusion of the intervention (Hevezi, 2016), the longer effects of these exercises on mindfulness have been inadequately addressed. The results of previous studies on nurses support that mindfulness training affects mental health positively but has no significant effect on physical health (Bazarko et al., 2013; Goodman & Schorling, 2012).

Social support refers to receiving emotional, intimate, material, and cognitive support from significant others (Williams et al., 2004). Social support from a supervisor has been correlated positively with compassion satisfaction and negatively with burnout and secondary traumatic stress (Hunsaker et al., 2015). Social support from relatives and friends has been found to moderate the negative effect of secondary traumatic stress on physical health (Fu et al., 2018). However, no study has examined the effect of social support on ProQOL. A social support intervention was found to significantly improve the physical health but not the mental health of nursing students at the end of the intervention (Walcott et al., 2018).

Few interventions have been developed to simultaneously improve ProQOL, physical health, and mental health in nurses. This type of intervention may require combining a variety of intervention components (Duhoux et al., 2017). Therefore, the purpose of this study was to examine the effects of an educational program that integrates compassion fatigue Resiliency, mindfulness Respiration, and Relatives and friends’ support (3R) to improve ProQOL, physical health, and mental health in nurses.

## Methods

### Design

A cluster experimental design was adopted to prevent contamination of the sample. Two teaching hospitals in southern Taiwan were selected and respectively assigned to either the experimental group or the control group. The participants in the experimental group received a 3R educational program in the hospital. The control group did not receive any intervention. Compassion satisfaction, burnout, secondary traumatic stress, physical health, and mental health were measured at baseline and at the end of the intervention (immediate effect), at 4 weeks postintervention (short-term effect), and at 12 weeks postintervention (medium-term effect). This study was conducted from May 2017 to December 2017.

### Participants

**Figure 1:**
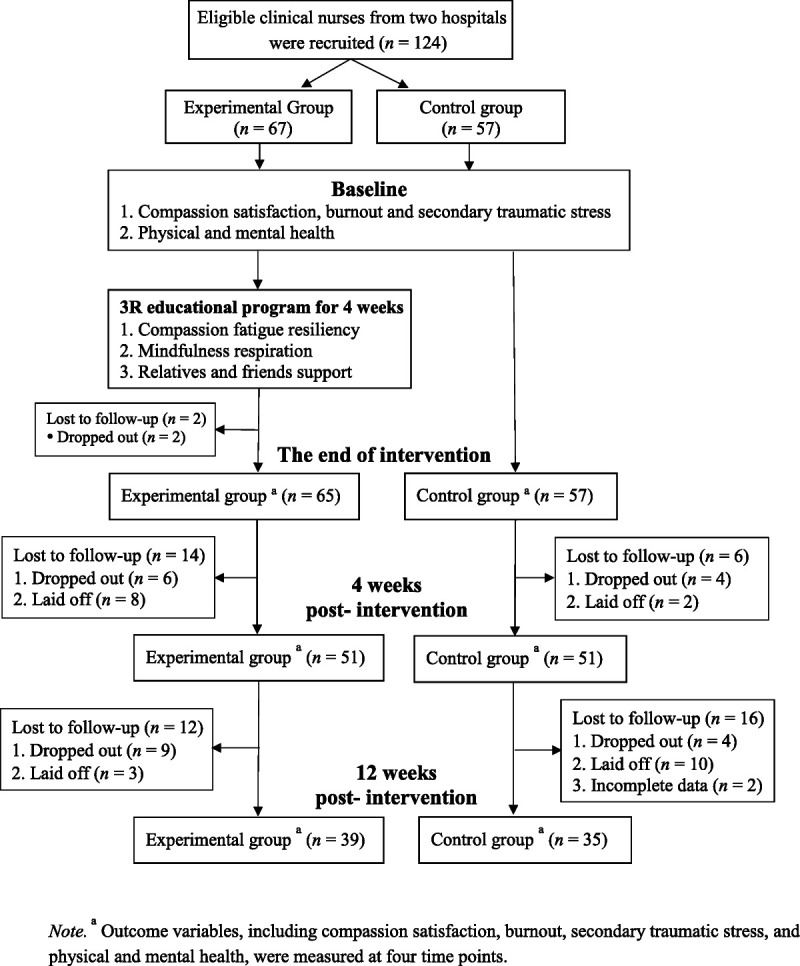
Flow of Study Recruitment, Intervention, and Measurements

The target population comprised clinical registered nurses. In this study, the inclusion criteria were registered nurses with at least 1 year of nursing experience. Eligible participants were recruited by the head nurses or nursing supervisors in the selected hospitals and referred to the researchers. After receiving signed consent forms from all of the participants, research assistants collected data from the two groups at baseline, at the end of the intervention, at 4 weeks postintervention, and at 12 weeks postintervention. Considering the lack of prior research on this topic, a medium effect size of 0.5 for the mean difference between the experimental and control groups was used for sample size estimation in this study (Cohen, 1988). Considering an effect size of 0.5, an alpha of .05, and a power of 0.8, 63 nurses were required for each group (Cohen, 1988). An initial group of 67 and 57 nurses were recruited for the experimental and control groups, respectively. The retention rates for the experimental group were 97.0% (*n* = 65) at the end of intervention, 76.1% (*n* = 51) at 4 weeks postintervention, and 58.2% (*n* = 39) at 12 weeks postintervention. The retention rates for the control group were 100% (*n* = 57) at the end of intervention, 89.5% (*n* = 51) at 4 weeks postintervention, and 61.4% (*n* = 35) at 12 weeks postintervention. The flow of recruitment, intervention, and measurement is shown in Figure 1.

### The 3R Educational Program Intervention

The compassion fatigue resiliency (Gentry et al., 2002) and mindfulness respiration (Kabat-Zinn, 1982) components of the 3R educational program were implemented by the researcher, whereas the relatives and friends’ support course component was conducted by a psychologist. The 3R educational program was implemented in once-per-week 2-hour sessions over a period of 4 weeks. The 3R educational program included several components, which are described in the following paragraphs.

#### Compassion fatigue resiliency

In the first week, the triggers and symptoms of compassion fatigue were introduced to the participants. Through self-evaluation, participants were taught to understand their risk levels for compassion fatigue and its adverse effects.

In the second week, self-regulation was introduced. Relaxation exercises were performed as self-regulation practice at the beginning of every class starting in the second week. Self-care and revitalization with an emphasis on better sleep and eating habits were also introduced in the second week.

In the third week, intentionality and perceptual maturation were introduced. Participants discussed the best solutions for dealing with stressful scenarios and learned to face negative situations, maintain positive mental perceptions, and avoid impulsive behaviors and reactions. Furthermore, self-validation was used to guide participants to practice talking themselves through the same stress scenario. Using self-validation, participants reflected on the appropriateness of different coping strategies. Participants were encouraged to apply appropriate strategies for managing similar, stressful situations in the future.

#### Mindfulness respiration

In the second week, the participants began practicing the breathing exercises associated with mindfulness respiration. In addition, the participants were encouraged to practice on their own and to record their exercise frequency and feelings in a notebook.

#### Relatives and friends’ support

In the fourth week, the participants were encouraged to join (or organize their own) support network. The participants were asked to think about whether they had a support network and to write down the style, members, and functions of this network in their notebook.

In the first week, the participants received a pamphlet containing self-evaluation forms, information regarding compassion fatigue, and a reminder note to practice mindfulness-respiration-related breathing exercises. Beginning in the second week, participants were asked to make a weekly record in their notebook, addressing what they had learned and their reflections on how they had applied the compassion fatigue resiliency components. These records were used as subjects for discussion in each of the following weeks.

### Measurements

A self-report questionnaire was used to collect the following data.

#### Personal characteristics

Personal characteristics data collected included age, education level, marital status, years of nursing experience, work setting, and manager (yes or no).

#### Physical and mental health status

The Chinese version of the SF-12v2 Physical and Mental Health Summary Scale (Ware et al., 1996) was used to assess the physical and mental health of the participants (six items each). The scale included a 3-point scale ranging from 1 (*extremely limited*) to 3 (*not limited at all*) for two items and 5-point scales ranging from 1 (*all of the time*) to 5 (*none of the time*) for eight items, 1 (*not at all*) to 5 (*extremely*) for one item, and 1 (*excellent*) to 5 (*poor*) for one item. The total scores for physical health and mental health ranged, respectively, from 6 to 26 and from 6 to 30. The original total scores for physical and mental health were standardized to create a possible score range between 1 and 100, with higher scores indicating better physical and/or mental health. In this study, the Cronbach’s alpha for physical health and mental health were .62 and .63 at baseline, respectively.

#### Professional quality of life

The Chinese version of the ProQOL Scale Version 5 (Stamm, 2010), including compassion satisfaction, burnout, and secondary traumatic stress (10 items each), was used to measure the ProQOL of participants. Each item was rated from 1 (*never*) to 5 (*always*). The five items of the burnout subscale were reverse-scored, and the possible total score for each subscale ranged from 10 to 50, with higher scores indicating higher levels of compassion satisfaction, burnout, or secondary traumatic stress. In this study, the Cronbach’s alpha values for compassion satisfaction, burnout, and secondary traumatic stress were .92, .82, and .82, respectively, at baseline.

### Ethical Issues and Approval

The institutional review boards of the experimental (IRB563B) and control (20150630B) hospitals approved the protocol for this study. All of the participants were informed that their participation in this study was voluntary, that all provided information would be kept confidential, and that they could decline to participate or withdraw at any time. All of the participants signed consent forms prior to participation.

### Statistical Analyses

IBM SPSS Statistics 21.0 (IBM, Inc., Armonk, NY, USA) was used to perform data analysis. Per protocol analysis was used in this study. A *t* test and a chi-square analysis were used to compare personal characteristics and outcome variables between the two groups at baseline, at the end of intervention, at 4 weeks postintervention, and at 12 weeks postintervention. Paired *t* tests were used to examine within-group differences from baseline to, respectively, the end of the intervention, 4 weeks postintervention, and 12 weeks postintervention. Generalized estimating equations were used to compare the differences in outcome variables between the experimental and control groups from baseline to the end of intervention, baseline to 4 weeks postintervention, and baseline to 12 weeks postintervention. Participants were considered to be random samples. A *p* value of less than .05 was considered statistically significant.

## Results

### Comparisons of Personal Characteristics and Outcome Variables Between the Experimental and Control Groups

As shown in Table 1, no significant difference in personal characteristics was indicated between the two groups. As shown in Table 2, compassion satisfaction, burnout, secondary traumatic stress, physical health, and mental health at baseline were all statistically similar between the two groups. Thus, the two groups were homogenous at baseline.

**Table 1:** Distributions of Personal Characteristics, With Comparisons Between Experimental and Control Groups (*N* = 124)

Variable	*n*	%	Experimental Group (*n* = 67)		Control Group (*n* = 57)		*t*/χ*^2^*	*p*
			*M*	*SD*	*M*	*SD*		
Age			39.01	9.27	37.09	8.53	1.20	.23
Years of nursing experience			16.71	9.07	15.06	8.94	0.95	.34
Work setting								
Ward	52	41.9	30	44.78	22	38.60	0.66	.72
Intensive critical unit	13	10.5	6	8.96	7	12.28		
Outpatient department	59	47.6	31	46.27	28	49.12		
Education level								
Junior college	23	18.5	13	19.40	10	17.54	0.07	.79
University	101	81.5	54	80.60	47	82.46		
Manager								
Yes	6	4.8	4	5.97	2	3.51	0.41	.52
No	118	95.2	63	94.03	55	96.49		
Marital status								
Single	44	35.5	21	31.30	23	40.40	1.09	.30
Married	80	64.5	46	68.66	34	59.65		

**Table 2:** Intergroup Comparisons of Compassion Satisfaction, Burnout, Secondary Traumatic Stress, and Physical and Mental Health Across Different Time Points

Variable	T0		T1		T2		T3	
	*M*	*SD*	*M*	*SD*	*M*	*SD*	*M*	*SD*
Compassion satisfaction								
Experimental	34.85	3.79	35.23	3.65	35.18	3.18	34.62	3.30
Control	32.94	4.45	30.74	4.95	28.80	4.29	27.31	3.64
*t*	1.97		4.95		7.74		8.90	
*p*	.06		< .001		< .001		< .001	
Burnout								
Experimental	30.62	4.07	27.13	3.47	28.77	3.79	28.62	3.40
Control	31.14	3.84	30.83	3.48	30.77	3.76	30.29	3.72
*t*	−0.57		−4.57		−2.27		−2.01	
*p*	.57		< .001		.03		.05	
Secondary traumatic stress								
Experimental	29.13	3.14	27.21	3.33	27.90	3.16	27.23	3.20
Control	29.49	3.66	28.86	4.42	29.69	4.20	29.37	4.07
*t*	−0.45		−1.84		−2.08		−2.49	
*p*	.66		.07		.04		.02	
Physical health								
Experimental	63.00	8.97	68.31	8.91	59.10	8.81	56.34	8.62
Control	59.00	10.15	54.78	10.01	54.64	10.31	53.93	12.68
*t*	1.78		6.11		1.96		0.96	
*p*	.08		< .001		.05		.34	
Mental health								
Experimental	52.82	7.93	64.87	9.08	58.95	8.25	55.82	8.34
Control	54.33	7.73	54.63	8.36	55.01	5.82	54.32	7.72
*t*	−0.83		5.05		2.39		0.80	
*p*	.41		< .001		.02		.42	

*Note.* T0 = baseline; T1 = end of intervention; T2 = 4 weeks postintervention; T3 = 12 weeks postintervention.

Compassion satisfaction was significantly higher in the experimental group than in the control group at the end of intervention, 4 weeks postintervention, and 12 weeks postintervention; burnout in the experimental group was significantly lower than in the control group at the end of intervention and 4 weeks postintervention; secondary traumatic stress in the experimental group was significantly lower than in the control group at 4 weeks and 12 weeks postintervention; physical health in the experimental group was significantly higher than in the control group at the end of intervention; and mental health in the experimental group was significantly higher than in the control group at the end of intervention and 4 weeks postintervention.

### Difference in Outcome Variables in the Experimental and Control Groups

As shown in Table 3, although compassion satisfaction did not significantly differ from baseline to the end of intervention, baseline to 4 weeks postintervention, or baseline to 12 weeks postintervention in the experimental group, it significantly decreased over all three time periods in the control group. Burnout significantly decreased from baseline to the end of intervention, baseline to 4 weeks postintervention, and baseline to 12 weeks postintervention in the experimental group but did not change significantly over these three time periods in the control group. Secondary traumatic stress significantly decreased from baseline to the end of intervention, baseline to 4 weeks postintervention, and baseline to 12 weeks postintervention in the experimental group and significantly decreased only between baseline and the end of intervention in the control group, with the baseline to 4 weeks postintervention and baseline to 12 weeks postintervention values unchanged. Physical health significantly increased from baseline to the end of intervention and significantly decreased from baseline to 4 weeks postintervention and baseline to 12 weeks postintervention in the experimental group, whereas it significantly decreased across all three time periods in the control group. Mental health significantly increased from baseline to the end of intervention, baseline to 4 weeks postintervention, and baseline to 12 weeks postintervention in the experimental group but did not significantly change over any of the three time periods in the control group.

**Table 3:** Within-Group Comparisons of Compassion Satisfaction, Burnout, Secondary Traumatic Stress, and Physical and Mental Health Across Postintervention Time Points

Variable	T1–T0		T2–T0		T3–T0	
	Pair *t*	*p*	Pair *t*	*p*	Pair *t*	*p*
Compassion satisfaction						
Experimental	1.38	.18	1.40	.17	−0.70	.49
Control	−9.23	< .001	−17.05	< .001	−14.88	< .001
Burnout						
Experimental	−9.60	< .001	−4.85	< .001	−5.31	< .001
Control	−1.11	.28	−0.97	.34	−2.13	.05
Secondary traumatic stress						
Experimental	−11.06	< .001	−3.83	< .001	−9.46	< .001
Control	−2.39	< .001	0.68	.50	−0.30	.77
Physical health						
Experimental	4.63	< .001	−2.62	.01	−4.97	< .001
Control	−5.24	< .001	−4.05	< .001	−5.68	< .001
Mental health						
Experimental	16.28	< .001	4.76	< .001	2.45	.02
Control	0.26	.80	1.02	.31	−0.10	.99

*Note.* T0 = baseline; T1 = end of intervention; T2 = 4 weeks postintervention; T3 = 12 weeks postintervention.

### Differences in the Changes in Outcome Variables Between Experimental and Control Groups

Personal characteristics data were adjusted using generalized estimating equations, with results showing the increases in level of compassion satisfaction in the experimental group from baseline to the end of intervention, baseline to 4 weeks postintervention, and baseline to 12 weeks postintervention to be significantly greater than those in the control group over the same time periods (Table 4). The decline in burnout in the experimental group from baseline to the end of intervention was significantly greater than in the control group. Furthermore, the decline in secondary traumatic stress in the experimental group from baseline to the end of intervention, baseline to 4 weeks postintervention, and baseline to 12 weeks postintervention was significantly greater than in the control group over the same time periods.

**Table 4:** Changes in Compassion Satisfaction, Burnout, Secondary Traumatic Stress, Physical Health, and Mental Health Between the Experimental and Control Groups Across Postintervention Time Points

Outcome Variable	β	*SE*	*p*	QIC	QICC
Compassion satisfaction				4,062.81	4,062.68
Intercept	29.52	2.65	< .001		
Group	1.68	0.87	.05		
Time			< .001		
Group × Time			< .001		
Group × Time (T1 vs. T0)	2.59	0.36	< .001		
Group × Time (T2 vs. T0)	4.48	0.34	< .001		
Group × Time (T3 vs. T0)	5.40	0.49	< .001		
Burnout				4,061.25	4,061.23
Intercept	36.00	2.88	< .001		
Group	−0.25	0.90	.78		
Time			< .001		
Group × Time			< .001		
Group × Time (T1 vs. T0)	−2.80	0.47	< .001		
Group × Time (T2 vs. T0)	−0.82	0.53	.13		
Group × Time (T3 vs. T0)	−0.69	0.53	.20		
Secondary traumatic stress				3,826.18	3,826.90
Intercept	31.16	2.32	< .001		
Group	−0.03	0.77	.97		
Time			< .001		
Group × Time			< .001		
Group × Time (T1 vs. T0)	−1.30	0.31	< .001		
Group × Time (T2 vs. T0)	−1.43	0.43	< .001		
Group × Time (T3 vs. T0)	−1.78	0.43	< .001		
Physical health				28,057.44	28,057.26
Intercept	65.48	6.51	< .001		
Group	4.67	2.15	.07		
Time			< .001		
Group × Time			< .001		
Group × Time (T1 vs. T0)	9.52	1.38	< .001		
Group × Time (T2 vs. T0)	0.46	1.81	.80		
Group × Time (T3 vs. T0)	−1.60	1.59	.31		
Mental health				1,834.71	18,347.81
Intercept	54.33	1.29	< .001		
Group	−1.51	1.80	.40		
Time			< .001		
Group × Time			.001		
Group × Time (T1 vs. T0)	11.74	1.37	< .001		
Group × Time (T2 vs. T0)	5.45	1.43	< .001		
Group × Time (T3 vs. T0)	3.02	1.42	.03		

*Note.* Personal characteristics were adjusted. QIC = quasi-likelihood under independence model criterion; QICC = corrected quasi-likelihood under independence model criterion; T0 = baseline; T1 = end of intervention; T2 = 4 weeks postintervention; T3 = 12 weeks postintervention.

Physical health improved significantly more in the experimental group than in the control group from baseline to the end of intervention. Also, the respective amounts of increase in mental health from baseline to the end of intervention, baseline to 4 weeks postintervention, and baseline to 12 weeks postintervention were significantly greater in the experimental group than in the control group.

## Discussion

Compassion satisfaction did not differ significantly from baseline to the end of intervention in the experimental group. This finding is in line with previous studies that applied compassion fatigue resiliency (Potter, Deshields, & Rodriguez, 2013) or mindfulness (Horner et al., 2014) interventions. Moreover, compassion satisfaction in the experimental group did not significantly increase from baseline to either 4 weeks or 12 weeks postintervention. Similarly, no significant effects on compassion satisfaction were found in prior compassion fatigue resiliency intervention studies at 2 months (Horner et al., 2014), 3 months (Potter, Deshields, Berger et al., 2013), or 6 months (Flarity et al., 2018) postintervention. Nevertheless, in this study, compassion satisfaction in the control group had significantly decreased during each of the three time periods. The 3R educational program in this study, though not improving compassion satisfaction, may have prevented its decline, which is consistent with the postintervention results at 3 weeks of a previous study that applied yoga and mindfulness programs (Gregory, 2015).

The different change patterns between baseline and the three postintervention time points between the experimental and control groups contributed to increasing compassion satisfaction in the experimental group significantly more than in the control group. Compassion fatigue resiliency and mindfulness respiration are highly related to the modification of negative perceptions, which may limit the improvement effect on compassion satisfaction. Positive psychological interventions that encourage people to develop positive emotions and perceptions toward events have been shown to improve well-being in nurses (Macfarlane & Carson, 2019). Further studies may integrate positive psychology interventions into the 3R educational program and examine the effect on improving compassion satisfaction.

Burnout in the experimental group significantly decreased from baseline to the end of intervention, which was consistent with previous studies that had used compassion fatigue resiliency (Potter, Deshields, & Rodriguez, 2013) and mindfulness interventions (Duarte & Pinto-Gouveia, 2016). The 3R educational program had an immediate and positive effect on burnout. In this study, burnout in the experimental group significantly decreased between baseline and both 4 and 12 weeks postintervention. Previous studies that had adopted only the compassion fatigue resiliency program reported finding no significant changes in burnout at 8 weeks (Flarity, Nash, et al., 2016) or 12 weeks (Potter, Deshields, Berger et al., 2013) postintervention. Social support was found to decrease burnout (Chou et al., 2014). The 3R educational program integrating social support may help sustain the reduction effects on burnout through the 4 and 12 weeks postintervention assessments.

Burnout in the experimental group increased significantly between the end of the intervention and 4 weeks (paired *t* = 3.48, *p* < .001) and 12 weeks (paired *t* = 3.68, *p* < .001) postintervention, respectively. Thus, although burnout decreased more significantly between baseline and the end of the intervention in the experimental group than in the control group, the intergroup difference was not significant for either the baseline-to-4-weeks or the baseline-to-12-weeks postintervention period. Burnout is highly associated with external stressors, such as excessive workload (Stamm, 2010). Therefore, the lack of improvement in external stressors may lead to the lack of short-term and medium-term effects of the 3R educational program on burnout.

Secondary traumatic stress in the experimental group had decreased significantly at the end of the 3R educational program, which is consistent with previous studies that had applied compassion fatigue resiliency interventions (Potter, Deshields, & Rodriguez, 2013) and mindfulness interventions (Duarte & Pinto-Gouveia, 2016). Secondary traumatic stress in the experimental group was also significantly lower at the 4-week and 12-week postintervention time points. This finding is similar to a previous study that found significantly lower secondary traumatic stress at 2 months after the completion of a compassion fatigue resiliency program (Flarity, Rhodes, et al., 2016). By contrast, secondary traumatic stress in the control group, though significantly lower at the end of intervention, was not significantly lower at the 4-week or 12-week postintervention time points. Therefore, declines in secondary traumatic stress were significantly greater in the experimental group than in the control group at the three postintervention time points. The 3R educational program had immediate, short-term, and medium-term ameliorating effects on secondary traumatic stress.

Physical health in the experimental group had significantly increased at the end of the intervention but had significantly decreased at the 4 weeks and 12 weeks postintervention. In this study, the 3R educational program had an immediate improvement effect on physical health, which differs from previous studies, which found no positive effect of mindfulness interventions on physical health (Bazarko et al., 2013; Goodman & Schorling, 2012). The physical health of the control group in this study decreased significantly at all three postintervention time points. Physical health may worsen over time in the absence of intervention. Differences in patterns of change between the two groups resulted in the experimental group having significantly better physical health than the control group at the end of the intervention. Social support interventions have been shown to positively affect physical health (Walcott et al., 2018). Support from relatives and friends may also moderate the negative effects of secondary traumatic stress on physical health (Fu et al., 2018). 3R educational programs that incorporate support from friends and relatives may provide a stronger, immediate effect on physical health in nurses.

Mental health in the experimental group had increased significantly at the end of the intervention, which is in line with the finding of prior studies that mindfulness interventions had short-term, improvement effects on mental health (Bazarko et al., 2013; Goodman & Schorling, 2012). The mental health of the experimental group at 4 weeks and 12 weeks postintervention had significantly increased over the baseline. Nevertheless, the mental health of the experimental group at 4 weeks (pair *t* = −4.33, *p* < .001) and 12 weeks (pair *t* = −6.07, *p* < .001) after the intervention was significantly lower than immediately after the end of the intervention. The 3R educational program had the most significant impact on mental health at the end of intervention. However, this effect diminished afterward. Because mental health scores in the control group remained unchanged across all of the three postintervention time points, the experimental group had significantly better mental health scores than the control group at each time point. The 3R educational program had immediate, short-term, and medium-term effects on improving mental health. Secondary traumatic stress is known to be associated more with mental health than with physical health (Fu et al., 2018). The demonstrated ability of the 3R educational program to ameliorate secondary traumatic stress may improve the mental health of nurses over the immediate, short, and medium terms.

### Limitation and Future Direction

The low retention rates in both the experimental (58.2%) and control groups (61.4%) at 12 weeks postintervention may be attributed to the time demand required to participate in the 4-week program. Developing online courses may be a feasible strategy to increase the retention rate. The results of a post hoc power analysis indicate that the intergroup powers of differences for secondary traumatic stress at the end of the intervention and for physical and mental health at 12 weeks postintervention were smaller than 0.8. The sample size in this study may have been insufficient to effectively examine the effects of the 3R educational program on secondary traumatic stress at the end of intervention and physical and mental health at 12 weeks postintervention. Furthermore, this study recruited participants from two hospitals in Taiwan only, which may have introduced a selection bias and limited the generalizability of the findings. Future studies should recruit nurses from a variety of hospitals and increase the sample size. In addition, the Cronbach’s alpha of the physical and mental health scale was relatively low in this study. Further studies should use scales to measure physical and mental health that offer better internal consistency. The three components of the 3R educational program were distributed evenly over the 4 weeks of the intervention and delivered in sessions lasting only 2 hours per week. The dose and frequency of the three components may not have been sufficient to demonstrate the short-term and medium-term effects on improving burnout and physical health. Future studies may increase the doses and frequencies of each component of the 3R educational program and examine their respective effects. In addition, the long-term effects of the 3R educational program on the ProQOL, physical health, and mental health of nurses should be assessed in future studies. We were unable to exclude the possibility of a Hawthorne effect in the experimental group, which may have overestimated the actual impact of the 3R educational program. Further studies may use a placebo group to more accurately examine the effect of the program.

### Conclusions

In this study, the 3R educational program was shown to provide positive effects on ProQOL, physical health, and mental health in the immediate term and to provide positive effects on compassion satisfaction, secondary traumatic stress, and mental health through the short and medium terms. The combined effects of the 3R educational program were better than the effects achieved by applying the components of 3R individually.

### Relevance to Clinical Practice

Nursing managers may apply a program in workplace settings that integrates compassion fatigue resiliency, mindfulness respiration, and relatives and friends’ support. The 3R educational program may be incorporated into on-the-job education programs. To overcome the challenges of shift work, this program may be distributed on compact disc or via asynchronous distance learning to achieve similar results. Finally, nursing managers should provide more resources to help clinical nurses build and sustain supportive groups of relatives and friends.
